# Efficacy and safety of fruquintinib dose‐escalation strategy for elderly patients with refractory metastatic colorectal cancer: A single‐arm, multicenter, phase II study

**DOI:** 10.1002/cam4.6786

**Published:** 2023-12-08

**Authors:** Sirui Tan, Shunyu Zhang, Nan Zhou, Xiaohong Cai, Cheng Yi, Hongfeng Gou

**Affiliations:** ^1^ Department of Medical Oncology, Cancer Center, West China Hospital Sichuan University Sichuan China; ^2^ Gastric Cancer Center West China Hospital, Sichuan University Sichuan China; ^3^ Sichuan Cancer Hospital & Institute, Sichuan Cancer Center, School of Medicine University of Electronic Science and Technology of China Chengdu China

**Keywords:** dose‐escalation, fruquintinib, geriatric assessment, mCRC, third‐line therapy

## Abstract

**Background:**

Fruquintinib has demonstrated significant improvement in overall survival (OS) among previously treated metastatic colorectal cancer (mCRC) patients. However, the utilization of fruquintinib has been constrained by various toxicities, such as hand‐foot skin reaction (HFSR) and hypertension, particularly in elderly patients with reduced tolerance to the standard dosage. This study aims to investigate the efficacy and safety of fruquintinib dose‐escalation strategy for elderly refractory mCRC patients.

**Patients and Methods:**

This open‐label, single‐arm, phase II trial included patients aged 65 years or over with mCRC who had progressed after two or more lines of chemotherapy. Fruquintinib was administered for 21 consecutive days of a 28‐day treatment cycle. The starting dose of fruquintinib was 3 mg/day and escalated to 4 mg/day in Week 2 and 5 mg/day in Week 3 if no significant drug‐related toxicity was observed. The highest tolerated dose from Cycle 1 would be administered in Cycle 2 and all subsequent cycles. Before commencing treatment, all enrolled patients underwent a G8 questionnaire and comprehensive geriatric assessments. The primary endpoint of the study was progression‐free survival (PFS).

**Results:**

A total of 29 patients were enrolled and all started fruquintinib at 3 mg/day. Fifteen patients (51.7%) were subsequently escalated to 4 mg/day and 4 (13.8%) to 5 mg/day. Only four (13.8%) patients discontinued treatment due to adverse events (AEs). The median PFS was 3.8 months (95% CI, 2.7–4.9), and the median OS was 7.6 months (95% CI, 6.5–8.7). Treatment‐related adverse events (TRAEs) were observed in all 29 patients (100%). The most frequently occurring (>10%) TRAEs greater than Grade 3 were HFSR (20.7%), hypertension (20.7%), and diarrhea (10.3%).

**Conclusion:**

Our study indicated that a dose of 4 mg/day was well tolerated by most elderly patients, suggesting that fruquintinib dose‐escalation strategy during the first cycle could serve as a viable alternative to the standard 5 mg/day dosing.

## 1 INTRODUCTION

Globally, colorectal cancer (CRC) ranks third in incidence and second in tumor‐related mortality among various malignant tumors in the world.^1^ Notably, approximately 60% of CRC patients are 65 years or older.^2^ However, clinical research evaluating drug therapies for CRC primarily enrolls patients under the age of 65. Consequently, new drug evaluation and evidence‐based medical data mainly come from younger patients. The proportion of elderly patients is relatively low in clinical trials, and those who are included typically exhibit favorable performance statuses and limited comorbidities.^3^, ^4^ This leads to uncertainty concerning efficacy, dose, and safety for elderly patients in clinical practice.

Fruquintinib is a potent and highly selective small‐molecule inhibitor of vascular endothelial growth factor receptor (VEGFR)‐1, ‐2, and ‐3 that inhibit tumor angiogenesis.^5^ The phase III FRESCO clinical study showed that fruquintinib in the third‐line treatment of metastatic CRC (mCRC) can prolong the survival of patients and reduce the risk of death by 35%.^6^ Approximately 18% (*n* = 50) of patients in the FRESCO study were patients aged 65 years or older. The standard daily dosage of fruquintinib, as established in the registration trial, was 5 mg/day, administered within a 3‐week‐on and 1‐week‐off regimen. Given that the FRESCO study reported an overall incidence of grade ≥3 adverse events (AEs) of 61.1% in the fruquintinib treatment group, and patients aged ≥65 exhibited a slightly elevated incidence of Grade 3/4 AEs in contrast to those below 65,^6^ there is a need to optimize the dosing strategy of fruquintinib in elderly patients with refractory mCRC.

In clinical practice, physicians often use various dose reduction or interval regimens to address the challenges faced by elderly patients who may exhibit poor tolerance to treatments, despite the lack of supportive clinical data. It is noteworthy that these strategies draw inspiration from the utilization of regorafenib. Regorafenib, an oral multitargeted kinase inhibitor, blocks several protein kinases involved in angiogenesis, oncogenesis, and tumor microenvironment.^7^ In the phase III CORRECT and CONCUR trial, regorafenib was shown to provide a survival benefit for mCRC patients in the third‐line treatment as well as fruquintinib. Multiple clinical studies of regorafenib have indicated that dose optimization or alternative regimens can improve drug tolerance and subsequently improve efficacy.^8^, ^9^ The ReDOS study indicated that the regorafenib dose‐escalation strategy in the first cycle tended to improve overall survival (OS) and reduce toxicity compared with the standard dose initiation regimen.^10^ Recognizing that elderly patients often manifest reduced tolerance in clinical practice, our study, leveraging insights from the ReDOS study, seeks to investigate the viability of fruquintinib dose‐escalation strategy, aimed at maintaining its antitumor efficacy while enhancing its tolerability.

The International Society of Geriatric Oncology has initially proposed that comprehensive geriatric assessment (GA) may help predict cancer treatment‐related toxicity and survival, as well as guide the choice and intensity of treatment.^11^ A full GA is time‐consuming. In contrast, the G8 questionnaire is a quick screening tool that has a high sensitivity for predicting abnormalities in the full GA. For the later‐line treatment of colorectal cancer, there is a scarcity of prospective studies investigating the association between GA and treatment outcomes. Notably, the G8 score has demonstrated potential as a predictive marker, as evidenced by the T‐CORE1401 study evaluating trifluridine/tipiracil (TAS‐102).^12^ Currently, there are no prospective studies on the GA of elderly patients with mCRC treated with third‐line antiangiogenic drugs.

Here, we report the efficacy and safety of fruquintinib dose‐escalation strategy for elderly refractory mCRC patients. Meanwhile, this study explored whether GA can predict the efficacy of fruquintinib treatment in elderly patients with mCRC.

## 2 METHODS

### 2.1 Patients and study design

This phase II prospective, single‐arm, open‐label, multicenter study was conducted at West China Hospital of Sichuan University, West China Fourth Affiliated Hospital of Sichuan University, Sichuan Cancer Hospital, and Sichuan Provincial People's Hospital. The study strictly adhered to the Helsinki provisions of the Declaration and obtained approval from the institutional review boards at each participating center. This trial is registered with ClinicalTrials.gov, number NCT05025631.

The key inclusion criteria were as follows: (1) patients aged 65 years or older who were diagnosed with mCRC confirmed by histopathology; (2) refractory patients defined the patients who progressed after two or more lines of standard chemotherapy (irinotecan, oxaliplatin, and fluorouracil) according to the Response Evaluation Criteria in Solid Tumors (RECIST) version 1.1; concurrent administration of VEGF or epidermal growth factor receptor (EGFR) inhibitors during chemotherapy was permitted; (3) Eastern Cooperative Oncology Group (ECOG) performance status of 0 or l; (4) able to take oral medication and expected survival time of at least 3 months; (5) presence of at least one measurable lesion in accordance with RECIST version 1.1; (6) informed consent and completed geriatric questionnaires at the time of enrollment; and (7) adequate organ and bone marrow function up to 2 weeks before enrollment.

The primary exclusion criteria included: (1) prior treatment with VEGFR inhibitors; (2) presence of central nervous system metastasis; (3) concurrent occurrence of uncontrolled hypertension, coronary artery disease, arrhythmia, heart failure, active hepatitis (defined as HBV DNA ≥10^3^ copies/ml after regular antiviral treatment), or congenital/acquired immune deficiency (such as HIV infection); (4) proteinuria of grade ≥2+ (equivalent to 1.0 g/24 h) level; and (5) multiple primary cancers.

### 2.2 Procedures

All included patients underwent GA and G8 screening tool before treatment. GA included Instrumental (IADL) and Activities in Daily Living (ADL) questionnaires, the Mini‐Nutritional Assessment (MNA), the Geriatric Depression Scale‐15 (GDS‐15), the Mini‐Mental State Examination (MMSE), the Cumulative Illness Rating Scale‐Geriatric (CIRS‐G) and Timed Up and Go (TUG) test.^13^ The G8 screening tool includes the MNA questionnaire and age.^14^

Fruquintinib was administered orally on 21 consecutive days in a 28‐day treatment cycle. All patients underwent dose escalation during the first cycle of fruquintinib treatment. The initial week involved oral administration of fruquintinib at a dosage of 3 mg/day. If this dosage was well tolerated, the dosage was increased to 4 mg/day during the second week. Subsequently, if the patient still exhibited tolerability, the dosage was further escalated to 5 mg/day during the third week. From the second cycle, patients were given the maximum dose that they had tolerated in the first cycle. CT scans were performed every two cycles for efficacy evaluation.

AEs were recorded following the National Cancer Institute's Common Terminology Criteria for Adverse Events (CTCAE) version 4.0. Adjustments to the fruquintinib dosage were made during treatment based on the occurrence of adverse events. If patients exhibited hand‐foot skin reaction, proteinuria, hypertension, decreased platelet count, bleeding, or abnormal liver function, dose modifications were implemented according to previously established criteria.^15^ In cases where other toxicities reached Grade 3 or higher, dose reduction or interruption was employed until the toxicity returned to Grade 1 or the baseline level. Attending physicians made the decision to suspend or discontinue drug administration as necessary. Re‐escalation of the dose was not recommended following dose reduction.

Criteria for patient withdrawal from the trial were as follows: (1) disease progression according to the (RECIST) 1.1 standard; (2) occurrence of unacceptable toxicity; (3) patient's request to withdraw from the trial; and (4) delay of more than 2 weeks in patient's treatment.

### 2.3 Outcomes

The primary endpoint was progression‐free survival (PFS) defined as the time from the start of treatment to the first verifiable disease progression or death due to cancer. Secondary endpoints were overall survival (OS), safety, and objective response rate (ORR). OS is defined as the time from the start of treatment to death from any cause. Tumor response was categorized as complete response (CR), partial response (PR), stable disease (SD), and progressive disease (PD). ORR was determined by evaluating patients who achieved either CR or PR.

Safety evaluation included AEs, laboratory tests, general physical examination, physical status score, electrocardiogram, etc. The investigator was responsible for taking corresponding treatment measures for AEs in patients.

### 2.4 Statistical analysis

In the phase II REGOLD study, the median PFS was 2.2 months. With a PFS threshold of 2.2 months and an expected PFS of 4.0 months, simulation results indicated a sample size of 26 with *α* = 0.05 (two sides) for a power of 80%, based on Power Analysis & Sample Size (PASS) statistical tool. Considering an estimated dropout rate of 10% among the participants, a target sample size of 29 was estimated.

Patient baseline characteristics were described. PFS and OS were estimated using Kaplan–Meier survival curves. The correlation between geriatric assessment and survival outcomes was evaluated by the Cox proportion hazard model. Statistical analyses were conducted using SPSS software. All statistical tests were two‐sided, and a significance level of *p* ≤ 0.05 was considered statistically significant.

## 3 RESULTS

### 3.1 Patient characteristics

Between November 6, 2020, and January 19, 2022, a total of 29 patients were enrolled in the study, and the cutoff date for OS was October 30, 2022. The median age of the patients was 69 years (range: 65–77), and 16 (44.8%) were male. Only 13.8% (4/29) of patients had an ECOG of 0. The primary tumor was located in the right colon in three cases (10.3%), in the left colon in 10 cases (34.5%), and in the rectum in 16 cases (55.2%). 79.3% (23/29) of patients underwent surgical resection of the primary tumor. Liver metastases (22/29) were the most common metastatic site. All patients received two or more previous chemotherapeutic regimens (median 2). Controllable hypertension (5/29; 17.2%) and type 2 diabetes (5/29; 17.2%) were the most common comorbidities. Most patients (27/29) had also received targeted therapy, with 82.8% (24/29) receiving bevacizumab and 10.3% (3/29) receiving anti‐EGFR monoclonal antibody. GA and G8 were performed for all patients at baseline. Detailed clinical characteristics, GA, and G8 information of the patients can be found in Table 1.

**TABLE 1:** Baseline clinical characteristics and geriatric assessment.

Baseline characteristics	*N* (%)
Age, years	
Median (range)	69 (65–77)
Sex	
Male	16 (55.2)
Female	13 (44.8)
ECOG performance status	
0	4 (13.8)
1	25 (86.2)
Primary tumor site	
Right colon	3 (10.3)
Left colon	10 (34.5)
Rectum	16 (55.2)
Metastatic site	
Liver	22 (75.9)
Lung	17 (58.6)
Surgery	
Yes	23 (79.3)
Comorbidities	
Hypertension	5 (17.2)
Type 2 diabetes	5 (17.2)
Others	19 (65.6)
History of target therapy	
Unused	2 (6.9)
VEGF inhibitors	24 (82.8)
EGFR inhibitors	3 (10.3)
Geriatric assessment *N* (%)	
G8	
≤14	20 (69.0)
>14	9 (31.0)
ADL	
≤5	1 (3.4)
>5	28 (96.6)
IADL	
≤7	9 (31.0)
>7	20 (69.0)
GDS‐15	
≤4	18 (62.1)
>4	11 (37.9)
MNA	
<24	26 (89.7)
≥24	3 (10.3)
TUG	
≤20	28 (96.6)
>20	1 (3.4)
MMSE	
>23	29 (100)

### 3.2 Treatment exposure and efficacy

**FIGURE 1:**
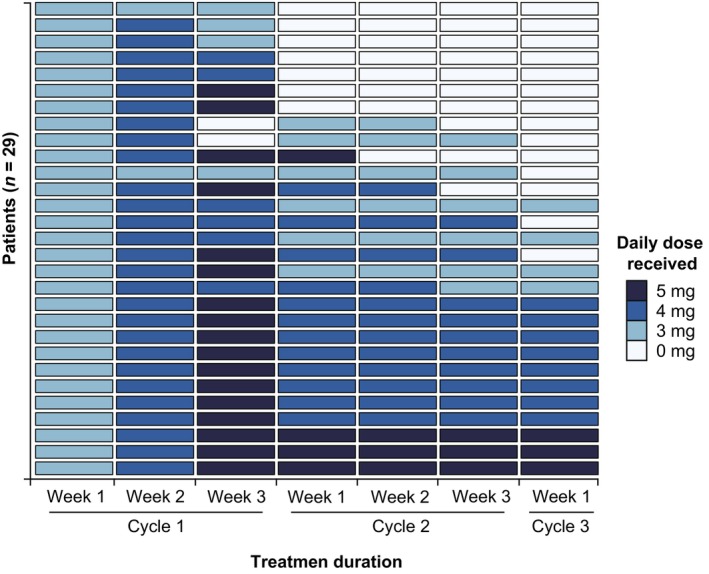
Swimmer plot presenting dosing history up to Week 1 of Cycle 3.

**FIGURE 2:**
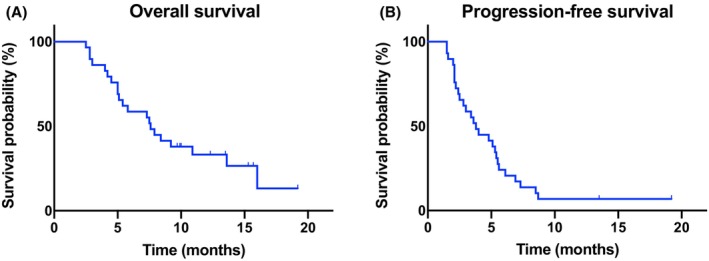
Survival outcomes with fruquintinib treatment. (A) The overall survival curve of the entire cohort. (B) The progression‐free survival curve of the entire cohort.

Among 29 patients, 34.5% (10/29) patients had a maximum tolerated dose of 3 mg fruquintinib, 51.7% (15/29) patients had a maximum tolerated dose of 4 mg, and only 13.8% (4/29) patients could tolerate a therapeutic dose of 5 mg. The weekly dosing history for patients up to Week 1 of Cycle 3 is summarized in Figure 1. Dose modifications (reduced, delayed, or interrupted) occurred in 26 (89.6%) of 29 patients. Dose omissions occurred in 11 (57.9%) patients. According to RECIST criteria, no CR or PR was observed. SD was observed in 16 patients (55.2%), and PD in 13 patients (44.8%; Table 2). After at least two cycles of treatment, the proportion of patients who started the third cycle of treatment was 51.7% (15/29). Disease progression (11/14) was the main reason for not starting the third cycle of treatment. Three patients for AEs reasons were not initiating a third cycle, among which one patient achieved SD and two achieved PD. As of October 30, 2022, 2 patients are still taking the drug, and 21 patients have died. The median PFS was 3.8 months (95% CI, 2.7–4.9) and the median OS was 7.6 months (95% CI, 6.5–8.7; Figure 2). Univariate analysis of 29 participants showed that sex, age, physical status, liver and lung metastasis, tumor site, surgery, and target therapy were not significantly associated with PFS or OS (*p* > 0.05; Tables 3 and 4). In geriatric assessment, TUG scores ≤20 s were more significantly associated with favorable PFS (*p* = 0.011 using the log‐rank test). However, only one patient with a TUG score >20 s in this trial, which could be biased.

**TABLE 2:** Maximum tolerable dose and efficacy with fruquintinib treatment.

	*N* (%)
Maximum tolerable dose	
3 mg	10 (34.5)
4 mg	15 (51.7)
5 mg	4 (13.8)
Therapeutic response assessment	
CR	0
PR	0
SD	16 (55.2)
PD	13 (44.8)
mPFS (95% CI)	3.8 (2.7–4.9)
mOS (95% CI)	7.6 (6.5–8.7)

**TABLE 3:** Univariate analysis of progression‐free survival correlations in 29 patients using a Cox proportion hazard model.

Characteristics	mPFS	HR (95% CI)	*p*‐value
Sex (female vs. male)	5.1 vs. 3.6	0.53 (0.24–1.19)	0.126
Age (>69 vs. ≤69)	5.1 vs. 2.7	0.68 (0.32–1.47)	0.332
PS (1 vs. 0)	3.6 vs. 5.5	1.19 (0.41–3.47)	0.754
G8 (≤14 vs. >14)	4.4 vs. 2.2	0.72 (0.32–1.63)	0.429
ADL (≤5 vs. >5)	5.5 vs. 3.7	0.79 (0.11–5.95)	0.821
IADL (≤7 vs. >7)	4.0 vs. 3.5	1.79 (0.74–4.33)	0.196
MMSE (>23)	3.8	‐	‐
GDS15 (>4 vs. ≤4)	4.8 vs. 2.9	0.76 (0.35–1.67)	0.497
MNA (<24 vs. ≥24)	3.9 vs. 3.4	0.70 (0.21–2.38)	0.572
TUG (>20 vs. ≤20)	1.5 vs. 3.9	39.24 (2.36–652.72)	0.011
Tumor site (right colon vs. none‐right colon)	2.5 vs. 3.9	0.96 (0.28–3.26)	0.949
Surgery (yes vs. no)	4.0 vs. 3.3	0.90 (0.36–2.28)	0.829
Liver metastasis (yes vs. no)	3.5 vs. 5.1	0.78 (0.32–1.94)	0.599
Lung metastasis (yes vs. no)	3.0 vs. 4.7	1.45 (0.66–3.16)	0.354

**TABLE 4:** Univariate analysis of overall survival correlations in 29 patients using a Cox proportion hazard model.

Characteristics	mOS	HR (95% CI)	*p*‐value
Sex (female vs. male)	7.5 vs. 8.0	1.00 (0.42–2.39)	0.993
Age (>69 vs. ≤69)	7.3 vs. 8.8	1.54 (0.62–3.82)	0.349
PS (1 vs. 0)	7.5 vs. NR	4.60 (0.61–34.88)	0.139
G8 (≤14 vs. >14)	7.4 vs. 7.9	1.20 (0.46–3.13)	0.713
ADL (≤5 vs. >5)	10.9 vs. 7.5	0.93 (0.12–7.05)	0.947
IADL (≤7 vs. >7)	5.8 vs. 7.8	1.08 (0.43–2.69)	0.873
MMSE (>23)	7.6	‐	‐
GDS15 (>4 vs. ≤4)	9.2 vs. 5.3	0.77 (0.32–1.83)	0.547
MNA (<24 vs. ≥24)	7.8 vs. 5.4	0.84 (0.19–3.67)	0.822
TUG (>20 vs. ≤20)	4.5 vs. 7.8	4.14 (0.5–34.45)	0.188
Tumor site (right colon vs. none‐right colon)	7.6 vs. 7.7	1.21 (0.28–5.28)	0.800
Surgery (yes vs. no)	8.4 vs. 5.9	0.43 (0.16–1.13)	0.088
Liver metastasis (yes vs. no)	7.8 vs. 7.3	0.92 (0.33–2.53)	0.867
Lung metastasis (yes vs. no)	7.5 vs. 8.1	1.06 (0.44–2.52)	0.903

### 3.3 Safety

Treatment‐related adverse events (TRAEs) were observed in all 29 patients (100%). Common AEs (>40%) included fatigue (23/29; 79.3%), decreased appetite (17/29; 58.6%), and hand‐foot syndrome (13/29; 44.8%). The most frequently occurring (>10%) TRAEs greater than Grade 3 were hand‐foot syndrome (6/29; 20.7%), hypertension (5/29; 17.2%), and diarrhea (3/29; 10.3%) as indicated in Table 5. Notably, patients experiencing Grade 3 or higher AEs accounted for 55.2% (16/29). Four patients (4/29; 13.8%) discontinued the treatment due to AEs, primarily attributed to fatigue (2/4) and hand‐foot syndrome (2/4). No Grade 5 TRAEs were observed.

**TABLE 5:** Treatment‐related adverse events.

Treatment‐related event, *n* (%)	Grades 1–2	Grades 3–4	Any grade
Fatigue	21 (72.4)	2 (6.9)	23 (79.3)
Decreased appetite	16 (55.2)	1 (3.4)	17 (58.6)
Hand‐food syndrome	7 (24.1)	6 (20.7)	13 (44.8)
Arthralgia	10 (34.5)	0	10 (34.5)
Muscle pain	9 (31.0)	1 (3.4)	10 (34.5)
Abdominal pain	9 (31.0)	0	9 (31.0)
Hoarseness	6 (20.7)	1 (3.4)	7 (24.1)
Mucositis oral	6 (20.7)	1 (3.4)	7 (24.1)
Hypertension	1 (3.4)	5 (17.2)	6 (20.7)
Diarrhea	3 (10.3)	3 (10.3)	6 (20.7)
Hemorrhage	5 (17.2)	1 (3.4)	6 (20.7)
ALT or AST elevation	5 (17.2)	0	5 (17.2)
Proteinuria	4 (13.8)	0	4 (13.8)
Vomiting	3 (10.3)	1 (3.4)	4 (13.8)
Leukopenia	2 (6.9)	0	2 (6.9)
Thrombocytopenia	2 (6.9)	0	2 (6.9)
Neutropenia	0	1 (3.4)	1 (3.4)

## 4 DISCUSSION

This study is the first to evaluate the efficacy and safety of fruquintinib initial dose‐escalation strategy for elderly patients with refractory mCRC. The mPFS in patients who were treated with this strategy was 3.8 months, and the primary reason for discontinuation of treatment was disease progression. The majority of elderly mCRC patients were able to tolerate a fruquintinib dosage of 4 mg/day, and fatigue was a major AE. Our study suggests that, for elderly patients with refractory mCRC, the fruquintinib dose‐escalation strategy could serve as a promising alternative to the standard 5 mg/day dosing.

In the phase III FRESCO study, the average age of the study participants was 54.6 years.^6^ The results demonstrated that the group treated with fruquintinib exhibited a significantly longer mOS compared with the placebo group (9.3 vs. 6.6 months, *p* < 0.001). Furthermore, the fruquintinib‐treated group demonstrated a significant improvement in mPFS compared with the placebo group (3.7 vs. 1.8 months, *p* < 0.001). In our current study, we specifically focused on elderly mCRC patients aged ≥65, with a median age of 69 years. We employed fruquintinib initial dose‐escalation strategy during the first cycle of treatment. The mPFS observed in our study was 3.8 months, while the mOS was 7.6 months. Although the mPFS in our study was similar to that reported in the FRESCO study (3.7 months), the mOS appeared to be shorter (9.3 months). One possible explanation for the lower overall survival observed in our study compared with the FRESCO study could be attributed to the difference in the proportion of patients who received targeted therapy as part of their front‐line treatment. In our study, a substantial majority (93.1%) of patients had previously received bevacizumab or cetuximab, whereas only 39.9% of patients in the treatment group of the FRESCO study had received targeted therapy. Previous studies have shown that mCRC patients who have received targeted drugs in the first and second lines can still derive benefits from third‐line treatments such as regorafenib and TAS102. However, it has been observed that the median overall survival (mOS) in patients receiving third‐line treatment is generally shorter compared to those who have not received targeted therapy before.^9^, ^16^ In a subgroup analysis of the FRESCO study, it was found that patients who had not received targeted therapy exhibited a longer mOS of 10.35 months when treated with fruquintinib, in contrast to patients who had previously received targeted therapy with a mOS of 7.69 months (95% CI, 6.90–10.09).^17^ The mOS in our study (7.6 months) was comparable to the OS outcomes from the FRESCO subgroup analysis, thereby corroborating the hypothesis of shorter OS associated with third‐line antitumor angiogenesis treatment of mCRC patients who had previously received targeted therapy.

In the present study, a dose‐escalation strategy was implemented during the first cycle of treatment, revealing that the majority of elderly patients with mCRC could tolerate a dose of 4 mg/day. Despite the lower intensity dose administered in this study compared with the overall population in the FRESCO study, the survival outcomes observed were similar. The FRESCO study has reported an incidence of 61.1% for grade ≥3 AEs, with 15.1% discontinuing treatment due to AEs.^6^ In our study, the incidence of grade ≥3 AEs was 55.2% (16/29), and 13.8% (4/29) of patients discontinued the drug due to AEs. Fatigue was one of the most common AEs observed in elderly patients in this study. In the prospective CONSIGN study and subgroup analysis of the CORRECT study, it was also shown that fatigue was common in elderly patients using regorafenib.^8^, ^18^ Collectively, the results of this study, along with existing data, suggest that severe fatigue may be the most common and limiting toxicity experienced by elderly mCRC patients receiving antiangiogenic small‐molecule drugs in third‐line therapy.

Although hypertension is a frequently reported AE of anti‐VEGF/VEGFR drugs as shown in this study and previously published literature,^19^ the blood pressure of the patients in this study was well controlled after receiving antihypertensive drugs, and no patient stopped fruquintinib permanently because of hypertension. Proteinuria, another known adverse reaction to VEGFR inhibitors, was relatively infrequent in this study, with only 13.8% (4/29) of patients experiencing mild proteinuria (Grades 1–2). This incidence was lower than what was reported in the FRESCO study (42.1%), which may be attributed to the utilization of lower doses in our study. No serious or fatal treatment‐related arterial/venous thromboembolic events, hypertensive crises, gastrointestinal perforation, or severe hepatotoxicity occurred in this study, although these serious AEs have been observed with other VEGFR inhibitors. Myelosuppression caused by fruquintinib was rare, as only 6.9% (2/29) and 3.4% (1/29) of patients in our study developed leukopenia and neutropenia, respectively.

Currently, limited research has been conducted to investigate the predictive value of GA in elderly colon cancer patients regarding the effectiveness of specific treatment regimens, and the findings have been controversial. A previous study has suggested that normal IADL is associated with better OS outcomes in elderly mCRC patients receiving first‐line therapy.^20^ In another study involving a cohort of 252 mCRC patients who received chemotherapy with or without bevacizumab, the G8 score was found to be significantly associated with PFS.^21^ However, the subgroup analysis of PRODIGE 20 showed no correlation between GA and G8 tools and the efficacy of chemotherapy with or without bevacizumab in elderly patients.^22^ For the later‐line treatment of colorectal cancer, there is a scarcity of prospective studies investigating the association between GA and treatment outcomes. Notably, the G8 score has demonstrated potential as a predictive marker, as evidenced by the T‐CORE1401 study evaluating trifluridine/tipiracil (TAS‐102).^12^ In this study, which included 30 patients with an average age of 73 years, higher G8 scores (>14 points) were associated with prolonged mPFS (4.6 vs. 2.0 months, *p* = 0.047) and mOS (9.3 vs. 3.9 months, *p* = 0.04) compared with lower G8 scores. However, at present, there are no prospective clinical studies to explore whether GA can predict the efficacy of patients with mCRC receiving third‐line antitumor blood vessel small‐molecule targeted drugs. Our study represents the first attempt to evaluate whether GA or G8 screening can serve as predictive tools for the efficacy of fruquintinib treatment. G8, ADL, IADL, MMSE, GDS‐15, and MNA were not associated with PFS or OS (*p* > 0.05). Nevertheless, a significant difference in PFS was observed among patients with TUG scores ≤20 and >20 (*p* = 0.01). However, the sample size of this study was small, which could be biased. Therefore, further investigations with larger sample sizes are warranted to explore the potential of G8 and GA in predicting the efficacy and tolerability of later‐line treatments in elderly patients with metastatic colorectal cancer.

Our study has several limitations, including a small sample size and a single‐arm design. Additionally, the comparison of data between our study and previous studies on regorafenib and TAS‐102 is challenging due to differences in research settings and studied populations. To provide more robust evidence, larger‐scale phase III clinical trials are necessary to validate the results obtained in this study.

## 5 CONCLUSIONS

Our study indicated that fruquintinib weekly dose‐escalation strategy during the first cycle maintained its efficacy and its safety profile was tolerable. Physicians may consider this fruquintinib dose‐escalation strategy as a viable alternative to the standard 5 mg/day dosing in the management of elderly patients with refractory mCRC.

## AUTHOR CONTRIBUTIONS

**Sirui Tan:** Data curation (equal); formal analysis (equal); methodology (lead); software (lead); visualization (equal); writing – original draft (equal); writing – review and editing (equal). **Shunyu Zhang:** Data curation (equal); investigation (equal); methodology (equal); writing – original draft (equal). **Nan Zhou:** Software (equal); writing – review and editing (equal). **Xiaohong Cai:** Project administration (equal); resources (equal); validation (equal). **Cheng Yi:** Investigation (equal); supervision (equal). **Hongfeng Gou:** Conceptualization (equal); investigation (equal); methodology (equal); project administration (equal); resources (equal); supervision (equal); writing – review and editing (equal).

## FUNDING INFORMATION

None.

## CONFLICT OF INTEREST STATEMENT

The authors have no conflict of interest.

## Data Availability

The data that support the findings of this study are available from the corresponding author upon reasonable request.
